# Comparing the strength of the confidence-accuracy versus response time-accuracy relationship for eyewitness identification

**DOI:** 10.1038/s41598-025-96224-y

**Published:** 2025-04-01

**Authors:** Curt A. Carlson, Robert F. Lockamyeir, Maria A. Carlson, Charles A. Goodsell, Alyssa R. Jones, Alex R. Wooten, Zane T. Brand

**Affiliations:** 1 East Texas A&M University, Commerce, TX USA; 2 State University of New York at Oneonta, Oneonta, NY USA; 3 Canisius University, Buffalo, NY USA; 4 Tarleton State University, Stephenville, TX USA; 5 Hollins University, Hollins, VA USA

**Keywords:** Eyewitness identification, Response time, Confidence, Memory strength, Showups, Lineups, Human behaviour, Long-term memory

## Abstract

Research indicates that eyewitness identification (ID) accuracy increases with faster IDs and those supported with immediate high confidence, but it is not clear which measure, confidence or response time, is the better reflector of accuracy. It is also important to know how well these patterns hold up across important factors affecting eyewitness ID accuracy such as memory strength for the perpetrator’s face. We conducted four pre-registered experiments to investigate these issues across different levels of target memory strength (via encoding time or image quality) and ID procedure (showups vs. lineups of different filler quality). Correct IDs were faster than false IDs regardless of memory strength, and this difference was greater for lineups than showups. There was a consistently strong positive CA relationship for those who made an ID, but the RTA relationship was significantly weaker. Both relationships were weaker for those who made a rejection decision, but the CA relationship remained stronger than the RTA relationship. We conclude that immediate confidence may be a more important reflector of accuracy than response time, regardless of the quality of the memory for the perpetrator’s face.

## Introduction

Despite decades of research indicating that eyewitness identification (ID) is highly problematic, there is now evidence that an initial high-confidence eyewitness ID from a well-conducted procedure, and in the absence of any memory contamination, can be highly accurate^1,2^. ID speed is also an important indicator of accuracy^3^. Recent research has shown that the combination of a fast ID with high confidence is most indicative of high accuracy^3^. However, our primary goal is to compare these measures separately because in many cases police do not have both pieces of information, regardless of current recommendations for best practices^4^. Therefore it is important to understand which measure may carry more value. It is arguably more straightforward for police to record confidence compared to decision time, and this may be one reason why it is much more common to collect confidence than decision speed^5^. For example, the showup (i.e., presenting a suspect by himself to a witness, rather than in a lineup) is still a popular ID procedure when a suspect is apprehended soon after a crime and in the vicinity, which police take as an opportunity to present the suspect to the eyewitness while their memory for the perpetrator is fresh^4^. They could walk him up to the witness, or drive him up in their police vehicle, but in such circumstances it is unclear how to accurately assess decision time, as there is no clear beginning and end to this ID procedure, unlike a photo lineup pulled from an envelope or presented via computer or tablet. Moreover, in the courtroom a jury may need to be educated about the value of confidence versus decision time, especially for cases in which only one measure of accuracy is available. For these reasons we conducted several experiments testing the value of both confidence and response time (RT) as *reflector variables*—behavior of the eyewitness at the time of the ID^4^—to measure eyewitness accuracy.

In doing so, we build upon the basic memory literature showing that both confidence and response latency are highly related to one another as well as to trace memory strength^6–8^. A recent paper focused on old/new recognition found that both confidence and response latency were related to discriminability^9^. In addition, they found that these relationships were stronger for “old” compared to “new” responses, which corresponds with the eyewitness memory literature finding stronger confidence-accuracy (CA) and response time-accuracy (RTA) relationships for those who identify a suspect (corresponding to an “old” response) compared to those who reject the ID procedure (corresponding to a “new” response).

More research on the eyewitness CA relationship is needed because recent research has revealed several exceptions to the *pristine conditions hypothesis*^1^. This hypothesis discounts the potential impact of suboptimal estimator variables (i.e., factors that occur at the time of the crime) on the CA relationship. There is theoretical support for why suboptimal estimator variables would not affect the CA relationship^10^, and this has been supported empirically (e.g., weapon presence^11^, cross race effect^12^, and encoding time^13^). In contrast, the optimality hypothesis^14,15^ states that conditions during a crime must be optimal in order to maintain a high CA relationship. When estimator variables are suboptimal, the CA relationship may suffer because of high confidence misidentifications. This pattern was recently supported by Giacona et al. [^16^; but see^17^], who compared a mock crime video with optimal encoding conditions versus a video with a combination of suboptimal conditions (e.g., greater viewing distance, disguise, visible weapon). Others have supported this finding with individual variables such as poor face recognition ability^18^, long distance between eyewitness and perpetrator^19^, poor sleep the night before the crime^20^, and a familiar innocent suspect^21^.

In continuation of this research, we will manipulate memory strength for the target. Recent research^22^ indicates that the effects of certain system variables (e.g., fair simultaneous lineup advantage over showups) are attenuated by increased encoding time. We seek to expand upon this research by investigating the potential impact of memory strength on the RTA relationship. Though the CA relationship is relatively unaffected by encoding time^1,13^, the RTA relationship has not been similarly tested. In addition, though the dynamics of the CA relationship are fairly well understood across ID procedures^1,23^ this is not the case for the RTA relationship.

The speed at which an eyewitness makes an ID is another reflector variable that is long known to be a strong indicator of accuracy: faster IDs are more accurate than slower IDs^3,12,24–31^. The underlying recognition memory theory is based on information accumulation^7,31^, such that a decision is made once sufficient evidence in favor of a particular outcome exceeds a criterion. For example, when an eyewitness views a lineup of six individuals, evidence differentially accumulates for each member. An accurate eyewitness likely has a stronger memory for the perpetrator; thus, evidence will quickly accumulate in the direction of a guilty suspect ID, rather than a filler ID or rejection. In contrast, an inaccurate eyewitness probably has a weaker memory for the perpetrator, thus making fillers in the lineup more competitive in accumulating information in their direction, which slows down RT. Signal Detection Theory (SDT)^32^ can also account for memory strength-based differences in RT^33^.

Brewer et al.^24^ manipulated memory strength via different retention intervals as well as lineup size. As expected, they found RT negatively correlated with accuracy for those who made an ID, but the manipulated variables changed the optimal time boundary for discriminating between accurate and inaccurate IDs, from 13 s with a 0 min retention interval to 36 s with a 15-min retention interval. Increasing lineup size (4 vs. 8 vs. 12) also increased RT, presumably due to the need to inspect more faces before making a decision. Key et al.^23^ found a strong RTA relationship for showups and fair lineups, but not biased lineups. One aspect of the current study is to expand upon these papers to demonstrate how important eyewitness ID variables may influence the RTA relationship.

## The present study

**Fig. 1:**
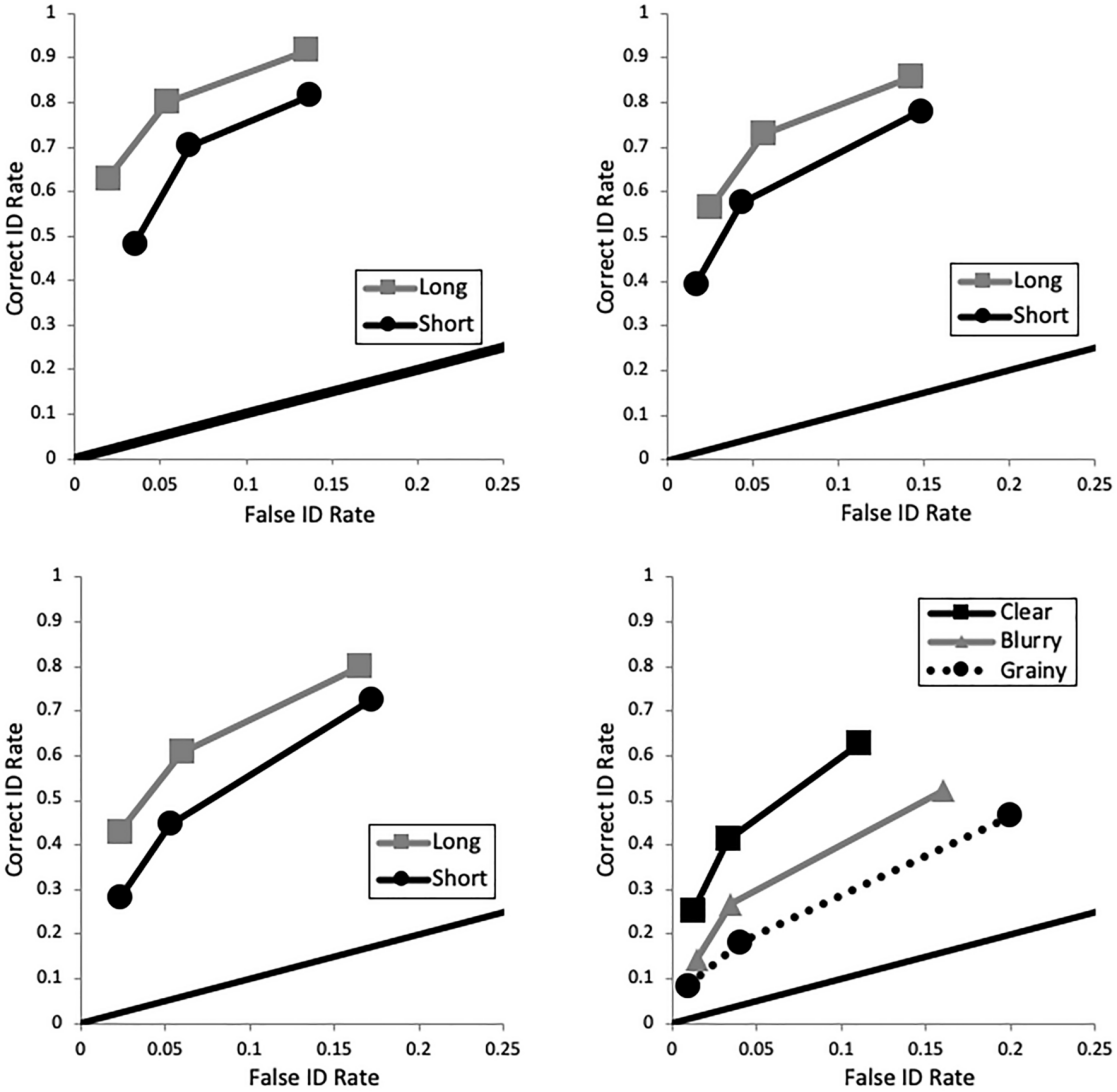
ROC curves depicting greater empirical discriminability with better target memory in the form of either longer encoding time (Pilot, upper-left; E1, upper-right; E2, lower-left) or clearer target image (E3, lower-right). “Long” indicates an encoding time of 5 s; “Short” indicates an encoding time of 1 s.

We first conducted a pilot experiment with only showups in order to test our experimental paradigm, hopefully avoiding floor and ceiling effects, as well as demonstrating typical CA and RTA relationships across our manipulation of memory strength. We pre-registered four hypotheses on the Open Science Framework (OSF): https://osf.io/wem46/files/osfstorage/6736326883e98dec3c07fa23. First, we expected that discriminability (*d*′) would be higher for faces encoded for 5 s compared to 1 s^13^. We will focus on *d*′ as our measure of discriminability, but we also generated Receiver Operating Characteristic (ROC)^34^ curves for all conditions across our experiments, which followed the same general pattern as the *d*′ results (these can be viewed on our OSF page, and we include some in Fig. 1). Second, we predicted that ID speed would be faster for faces encoded for 5 s compared to 1 s. This prediction is based on the diffusion model^7,8^ (when memory strength is manipulated within-subjects as we describe below), SDT^33^, as well as Brewer et al.^24^ results showing slower identifications with increased retention interval (i.e., lower memory strength). Third, based on the *pristine conditions hypothesis*^1^, we predicted a positive CA relationship regardless of encoding time. Lastly, we expected a negative RTA relationship regardless of encoding time.

## Pilot experiment

### Method

#### Participants

Using G*Power 3.1^35^, we determined that a sample size of 50 would be sufficient to achieve 0.80 power to detect an effect size (*η*_*p*_^2^) of 0.17 from a repeated-measures MANOVA. We slightly exceeded this goal with *N* = 52 undergraduate psychology students from a small southern U.S. university as participants. The demographic breakdown of the sample is as follows: (a) sex: 71% female; (b) age: 84% between 18 and 24, 9% between 25 and 30, and 7% between 30 and 40; (c) race/ethnicity: 56% Caucasian, 17% African-American, 17% Hispanic or Latino, 6% Asian or Indian, and 4% other or chose not to report.

#### Stimuli

The face stimuli (all young adult Caucasian males) came from three face databases: Amsterdam Dynamic Facial Expression Set (ADFES)^36^, Karolinska Directed Emotional Faces (KDEF)^37^, and Radboud Faces Database^38^. We utilized these databases because they contained an angry and neutral expression for each identity, and we wanted our participants to study an angry expression and be tested with a neutral expression. It is important to present a different picture of a face between study and test in order to assess face memory rather than picture memory^39^. All experiments were presented with E-Prime 3.0^40^, allowing us to control stimuli presentation and record keyboard responses along with response time.

#### Design and procedure

We used a within-subjects design: 2 (encoding time for each target face: 1 s or 5 s) × 2 (target-present or -absent showup), with each participant taking part in 40 blocks (10 for each condition). Eyewitness ID data from a multiple-blocks design tend to mimic data from single trial eyewitness ID experiments^41^. Each block contained: (a) A target face presented with an angry expression for either 1 s or 5 s; (b) working on word-search puzzles for one minute; (c) a warning that the upcoming test face may or may not be the target face shown previously in that block^42^; (d) a yes/no identification decision for a showup (neutral expression), which was either the same target just presented in the same block, or a new face; (e) confidence assessment on a 0–10 scale, with 0 = 0% confident in the decision, and 10 = 100% confident in the decision. The order of faces was randomized, and encoding time and target presence were counterbalanced across blocks. Each participant was seated at a PC workstation in a separate cubicle, and was not able to view other participants or their computers. Anywhere from 1 to 4 students could participate in the lab at a given time, with the experiment lasting 45–60 min. Each participant contributed 10 data points per cell of our 2 × 2 within-subjects design, resulting in 520 data points per cell (based on *N* = 52). We needed this number in order to create stable confidence-accuracy characteristic (CAC) and response time-accuracy characteristic (RAC) curves based on the eyewitness literature^3,43^. Both CAC and RAC curves plot suspect ID accuracy (correct ID rate/[correct + false ID rate]) on the y-axis, but the former has confidence on the x-axis whereas the latter has RT (or equivalent, like Log RT) on the x-axis.

### Results

Table 1 contains all decisions across experiments and conditions. When *d*′ values are reported below (for showups or lineups), they are based on *d*′ = *z*(HR)–*z*(FAR)^44^ for each participant’s HRs and FARs from a given condition, and then averaged across those *d*′ values for that condition. For both showups and lineups, HR = correct ID rate for guilty suspects, and FAR = false ID rate for innocent suspects (not fillers). In order to avoid multicollinearity, we did not combine these variables in a MANOVA model. Instead, we applied an individual dependent *t*-test to each measure and controlled for increases in Type I error rate with Bonferroni correction to alpha (0.5/3 = 0.0167). All reported *p*-values are two-tailed. Discriminability was greater for longer (3.19) compared to shorter encoding (2.61), *t*(51) = 3.04, *p* = 0.004, Cohen’s *d* = 0.42. This difference in *d*′ was driven by higher correct ID rate with longer (0.92) over shorter encoding time (0.82), *t*(51) = 4.33, *p* < 0.001, *d* = 0.60. There was no difference in false ID rate (short = long = 0.14), *t*(51) = 0.12, *p* = 0.91.

**Table 1:** Identification and rejection data across experiments.

Experiment	Target encoding time or clarity	Procedure	Target-present			Target-absent		
			Correct ID rate	Filler ID rate	Rejection rate	False ID rate	Filler ID rate	Rejection rate
Pilot	1 s	Showups	0.82 (372/456)	N/A	0.18 (84/456)	0.14 (70/509)	N/A	0.86 (439/509)
	5 s		0.92 (476/519)	N/A	0.08 (43/519)	0.14 (63/465)	N/A	0.86 (402/465)
1	1 s	Showups	0.75 (343/459)	N/A	0.25 (116/459)	0.10 (48/459)	N/A	0.90 (411/459)
		Biased lineups	0.81 (410/504)	0.06 (30/504)	0.13 (64/504)	0.23 (118/504)	0.16 (81/504)	0.61 (305/504)
		Fair lineups	0.78 (374/477)	0.08 (39/477)	0.13 (64/477)	0.10 (50/477)	0.20 (96/477)	0.69 (331/477)
	5 s	Showups	0.85 (391/459)	N/A	0.15 (68/459)	0.12 (55/459)	N/A	0.88 (404/459)
		Biased lineups	0.88 (442/504)	0.05 (24/504)	0.08 (38/504)	0.18 (90/504)	0.12 (58/504)	0.71 (356/504)
		Fair lineups	0.84 (403/477)	0.06 (30/477)	0.09 (44/477)	0.13 (61/477)	0.15 (70/477)	0.73 (346/477)
2	1 s	Showups	0.71 (180/252)	N/A	0.29 (72/252)	0.19 (48/248)	N/A	0.81 (200/248)
		Biased lineups	0.69 (232/336)	0.06 (19/336)	0.25 (85/336)	0.19 (64/332)	0.13 (42/332)	0.68 (226/332)
		Fair lineups	0.77 (239/309)	0.06 (19/309)	0.17 (51/309)	0.13 (41/312)	0.25 (79/312)	0.62 (192/312)
	5 s	Showups	0.78 (194/249)	N/A	0.22 (55/249)	0.16 (40/249)	N/A	0.84 (209/249)
		Biased lineups	0.79 (266/335)	0.04 (13/335)	0.17 (56/335)	0.17 (56/332)	0.11 (36/332)	0.72 (240/332)
		Fair lineups	0.82 (258/313)	0.04 (12/313)	0.14 (43/313)	0.17 (52/313)	0.21 (66/313)	0.62 (195/313)
3	Clear	Showups	0.72 (385/534)	N/A	0.28 (149/534)	0.15 (79/534)	N/A	0.85 (455/534)
		Lineups	0.53 (279/522)	0.14 (71/522)	0.33 (172/522)	0.07 (38/522)	0.21 (110/522)	0.72 (374/522)
	Blurry	Showups	0.66 (351/534)	N/A	0.34 (183/534)	0.25 (131/534)	N/A	0.75 (403/534)
		Lineups	0.39 (201/522)	0.24 (124/522)	0.38 (197/522)	0.07 (39/522)	0.35 (183/522)	0.57 (300/522)
	Grainy	Showups	0.58 (312/534)	N/A	0.42 (222/534)	0.28 (149/534)	N/A	0.72 (385/534)
		Lineups	0.34 (180/522)	0.28 (144/522)	0.38 (198/522)	0.12 (63/522)	0.34 (178/522)	0.54 (281/522)

We also applied ROC analysis, which is a more objective, theory-free method for demonstrating differences in discriminability^34^. We constructed ROC curves by plotting correct and false identification rates at varying confidence levels. The rightmost point on a curve represents all IDs regardless of confidence level, and then the curve is drawn to the left by increasing response criterion by dropping IDs with lower levels of confidence. Specifically, the second point from the right contains IDs supported by 30–100% confidence, then the next point represents IDs supported by 50–100% confidence, and so forth until the leftmost point contains IDs supported by 90–100% confidence (i.e., the most conservative IDs). Empirical discriminability is determined by partial area under each curve (pAUC), and we compared each pair of pAUCs with the pROC statistical package^45^. The curves are partial because they do not include all possible decisions, such as rejections and filler IDs (from lineups). Instead, they focus on arguably the most important eyewitness decisions: suspect IDs. The specificity (1—FAR) for each comparison was based on the max FAR in the comparison to prevent loss of data. As shown in Fig. 1 (upper-left panel), 5 s yielded greater discriminability (pAUC = 0.116) compared to 1 s encoding time (pAUC = 0.091), *D* = 3.51, *p* < 0.001.

Turning to RT (range from Mdn 2100 ms [correct IDs after short encoding]—Mdn 4320 ms [false IDs after long encoding]), we first normalized with a log transformation^46,47^, followed by elimination of all outliers beyond 2 SD from the mean (approximately 4% of data)^48^. We then applied an ANOVA model to determine whether correct IDs were faster than false IDs, and whether encoding time made a difference. Correct IDs (*M* = 3.37) were faster than false IDs (*M* = 3.57), *F*(1, 977) = 104.73, *p* < 0.001, *η*_*p*_^2^ = 0.097, but there was no difference based on encoding time (contrary to our second hypothesis), *F*(1, 977) = 3.01, *p* = 0.083, *η*_*p*_^2^ = 0.003, and no interaction, *F*(1, 977) = 0.26, *p* = 0.61.

**Fig. 2:**
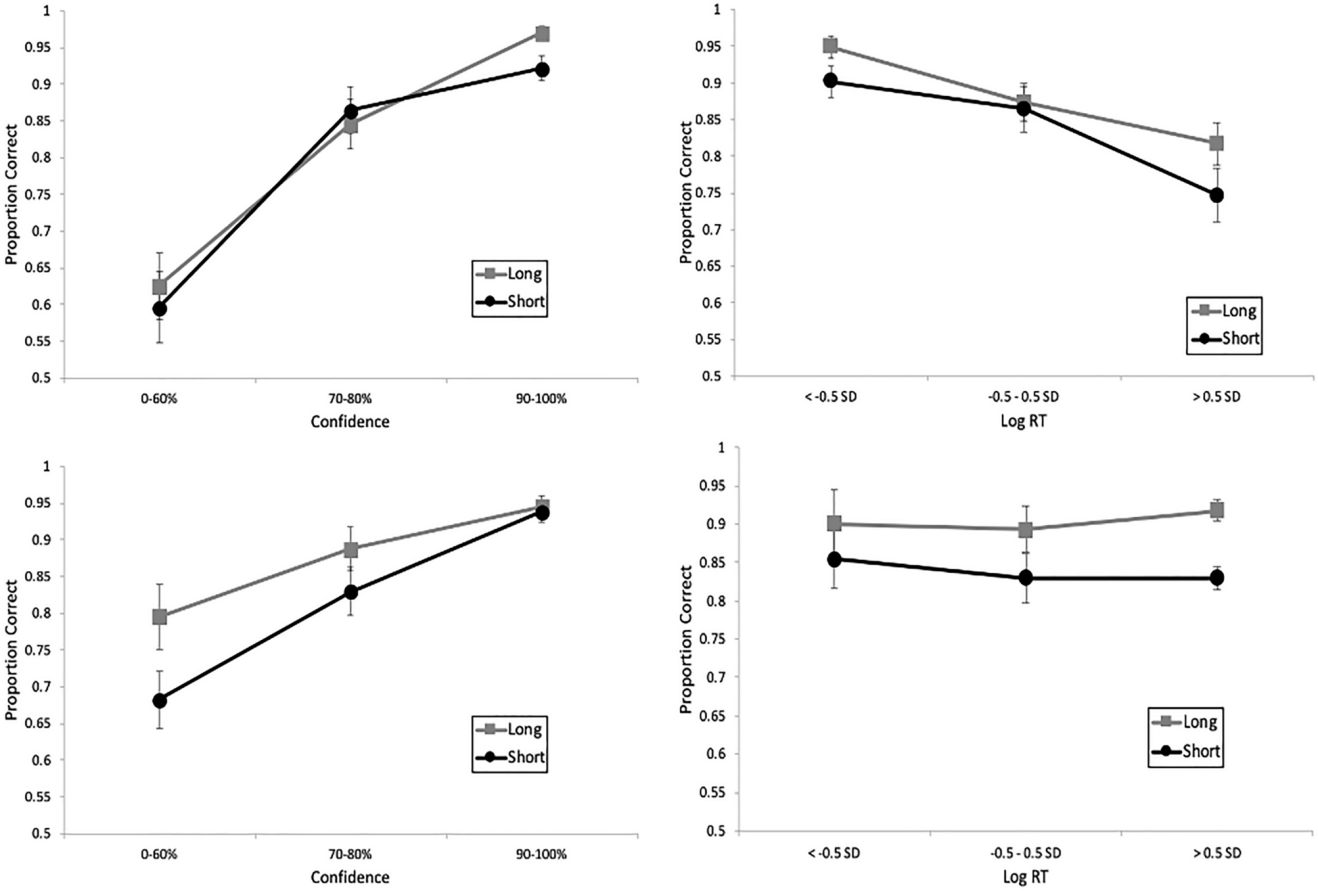
CAC (left panels) and RAC curves (right panels), with standard error bars, from the pilot experiment. In the top row, proportion correct is based on identifications (correct/[correct + false IDs]), and in the bottom row it is based on rejections (correct rejections/[correct rejections + incorrect rejections]). “Long” indicates an encoding time of 5 s; “Short” indicates an encoding time of 1 s.

Figure 2 (top left panel) depicts a strong CA relationship for IDs regardless of encoding time, in support of our third hypothesis. However, longer encoding did yield higher accuracy (0.97) than shorter encoding (0.92) at the highest level of confidence, *z* (2-tailed) = 2.7, *p* = 0.007. In the bottom left panel of Fig. 2 are the CAC curves based on rejections, which also portray a positive CA relationship, though noticeably flatter than for IDs.

The RAC curves are presented on the right of Fig. 2, with the fastest RTs on the left and the slowest RTs on the right. The x-axis is based on the normal distribution of log RT, with the fastest responses on the left (faster than 0.5 SD below the mean; about 31% of the distribution), average speed responses in the middle 1 SD (about 38% of the distribution), and the slowest responses on the right (slower than 0.5 SD above the mean; about 31% of the distribution). We took this approach not only to normalize the distribution but also to ensure a sufficient number of data points in each bin for comparison across RAC bins and also between RAC and CAC bins. One of the primary reasons why CAC curves are binned as low confidence (0–60%), medium confidence (70–80%), and high confidence (90–100%) is to include a sufficient amount of data in each bin^49^, and we took the same approach with the RAC graphs. We also investigated our RAC curves based on raw RT and with several different bins (e.g., < 2 s for fast and > 6 s for slow; < 3 s for fast and > 5 s for slow), which did not change the results.

Similar to the CA relationship, encoding time did not affect the RTA relationship for IDs (top right panel), though longer encoding yielded marginally higher accuracy (0.95) than shorter encoding (0.90) at the fastest RT bin, *z* (2-tailed) = 1.87, *p* = 0.06, similar to the CA pattern at the highest level of confidence. Interestingly, the RAC curves are noticeably flatter than the CAC curves for both IDs and rejections, implying that there is more information about accuracy based on confidence compared to RT. This provides somewhat mixed evidence for our fourth and final hypothesis, as we expected a relatively strong RTA relationship (for IDs) regardless of encoding time, but also thought it would be more equivalent to the CA relationship in terms of strength. In sum, confidence reflected accuracy regardless of whether participants identified or rejected suspects presented in showups, but RT reflected accuracy more weakly for IDs and not at all for rejections.

In Table 2 we present two measures of the value of CACs and RACs in reflecting accuracy: (a) accuracy range covered by the reflector, and (b) *z*-score comparing the highest and lowest points of that range as an indication of significance as well as effect size. From these measures we draw two conclusions: (a) confidence and RT are better reflectors of accuracy for IDs compared to rejections, and (b) confidence is a consistently better reflector of accuracy compared to RT.

**Table 2:** Three statistical indices to compare CAC and RAC curves.

Experiment	Type of curve	Type of response	Accuracy range	*z*
Pilot	CAC	IDs	0.61–0.95	12.16**
		Rejections	0.73–0.94	7.92**
	RAC	IDs	0.78–0.93	5.71**
		Rejections	0.86–0.88	0.77
Experiment 1	CAC	IDs	0.64–0.96	19.99**
		Rejections	0.77–0.92	9.07**
	RAC	IDs	0.78–0.91	7.24**
		Rejections	0.83–0.87	1.79
Experiment 2	CAC	IDs	0.66–0.93	12.42**
		Rejections	0.74–0.81	3.03*
	RAC	IDs	0.74–0.86	4.93**
		Rejections	0.76–0.77	0.38
Experiment 3	CAC	IDs	0.69–0.93	10.93**
		Rejections	0.60–0.73	6.44**
	RAC	IDs	0.76–0.79	1.29
		Rejections	0.64–0.68	2.04*

CAC, confidence-accuracy characteristic; RAC, response time accuracy characteristic; IDs, identifications. *Significant at 0.05 level. **Significant at 0.001 level. *z* is 2-tailed and based on comparison of the highest and lowest proportions in the accuracy range.

Another way to demonstrate the stronger CA relationship compared to the RTA relationship is to conduct mixed model analyses^50^ that treat participants and trials as random effects and our manipulations as fixed factors, thereby controlling for individual differences and dealing with confidence, RT, and LogRT as continuous measures without the need for binning in CAC or RAC space. We tested three such models, each of which focused on a different reflector: confidence, raw RT, and Log RT. In support of the CAC and RAC analyses, the *F*-values from the confidence model exceeded the *F*-value associated with the RT or Log RT model: (a) confidence model: *F*(1, 1945) = 248.44, *p* < 0.001; (b) raw RT model: *F*(1, 1945) = 66.25, *p* < 0.001; (c) LogRT model: *F*(1, 1945) = 63.23, *p* < 0.001.

### Discussion

Longer encoding time boosted discriminability and correct IDs were made faster than false IDs, but we also expected faster decisions after longer encoding time, which was not supported. As predicted, there was a positive CA relationship and a negative RTA relationship for IDs^51^, but the negative RTA relationship for IDs was weaker than expected. One possible reason for these unexpected results is that the task was too easy, based in part on the distractor task (word-search puzzle) participants worked on for one minute between each target face and showup. Correct ID rate after long encoding time approached ceiling (0.92), and false ID rate, regardless of encoding time, was low (0.14). One goal for our first primary experiment was to increase task difficulty.

## Experiment 1

Our pilot results are based only on showups, but lineups are also commonly used by police and are currently recommended over showups^4^. While showups have largely been found to reduce accuracy compared to lineups^52^, this pattern does not always emerge^53–55^, particularly for multi-block experiments^56^. Therefore, our predictions below regarding differences between showups and lineups were somewhat tentative. Our primary three experiments included showups along with two types of simultaneous lineups: biased and fair. Key et al.^23^ compared showups with biased and fair lineups, but their biased lineups were extremely biased toward the suspect, as the fillers did not match broad characteristics of the perpetrator such as age. We sought to create lineups more likely to be used by police, though with a significant difference between more biased and more fair lineups in terms of the similarity relationship between fillers and suspect. We accomplished this goal (see link below to supplemental document describing lineup creation and fairness assessment), but we acknowledge that our fair lineups were not perfectly fair because each target-absent lineup contained a designated innocent suspect chosen based on looking similar to the target in the associated target-present lineup. This experiment was also pre-registered on OSF: https://osf.io/62kvf/?view_only=4959cf8208b5419d9e6c55624434c643

We added the following predictions to the original four: stronger CA and RTA relationship, and slower ID speed, for fair lineups compared to biased lineups or showups.

### Method

#### Participants

A new power analysis with G*Power provided our goal of at least 90 participants (i.e., at least 30 per between-subjects condition; see design below) in order to achieve 0.80 power to detect an effect size (*η*_*p*_^2^) of 0.18 from a mixed-effects MANOVA. We ultimately obtained data from *N* = 136 undergraduate psychology students across two universities: the same small southern U.S. university as the pilot, and also a small university in the northeast U.S. This allowed us to detect smaller effect sizes (*η*_*p*_^2^ = 0.12). The demographic breakdown of the sample is as follows: (a) sex: 76% female; (b) age: 87% between 18 and 24, 13% between 25–30; (c) race/ethnicity: 61% Caucasian, 13% Hispanic or Latino, 11% African-American, 8% Asian or Indian, and 7% other or chose not to report. All experiments in this paper were approved by the Institutional Review Board at East Texas A&M University, in accordance with relevant guidelines and regulations, and we received informed consent from all participants.

#### Stimuli

**Fig. 3:**
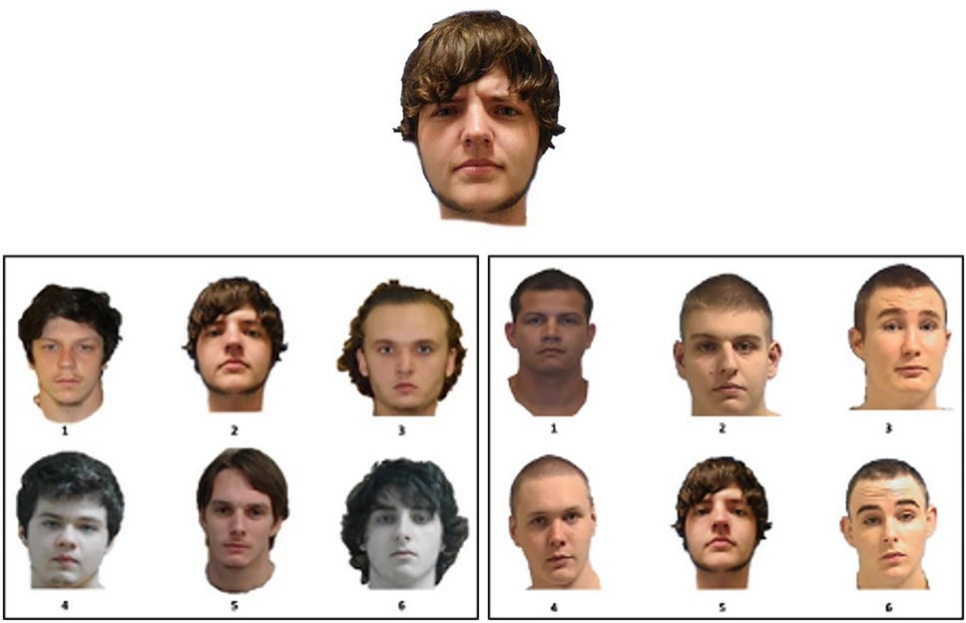
Example target (top), fair lineup (left), and biased lineup (right) from Experiments 1 and 2.

Our targets (all young adult Caucasian males) came from a new face database for Experiments 1 and 2: Selfies for Science^57^ (used with permission), which like the databases used in the pilot had an angry and neutral version of each identity so that we could present the former at study and the latter at test. We again presented the experiment with E-Prime 3.0^40^. The computer-generated faces presented as the distractor task in each block were created with FACES 4.0^58^. Lineup fillers came from several publicly available prison databases (e.g., State of Kentucky, State of Michigan). With the targets coming from a face database and fillers coming from prison mugshots, we were careful to modify the targets to be of similar image quality as the fillers (see supplemental material for a description of how we created lineups here: https://osf.io/wem46/files/osfstorage/6736325058ecea85c376bef4). See Fig. 3 for an example target (angry expression) and associated TP fair and biased lineup.

#### Design and procedure

This experiment featured a mixed design: 2 (1 vs. 5 s encoding, within subjects) × 3 (showup, biased lineup, fair lineup, between subjects) × 2 (target-present or -absent ID procedure, within subjects). Each participant took part in 36 blocks, meaning they provided an ID decision from 36 lineups. Based on our two within-subjects IVs with two levels each (encoding time of 1 vs 5 s; TP/TA lineup), these 36 are divided by 2 × 2 = 4, resulting in nine data points per participant. Half of our participants viewed 5 TP lineups and 4 TA lineups, with the other counter-balanced half viewing 4 TP lineups and 5 TA lineups. These allowed us to calculate a correct ID rate (from the 4–5 TP lineups) and false ID rate (from the 4–5 TA lineups) from each participant, then *d’* was calculated based on the combined correct and false ID rates. Each block contained several parts:

1. A target face was presented with an angry expression for either 1 or 5 s.
2. A new distractor task occurred for 1 min, during which they provided attractiveness ratings for each of six computer-generated faces (one per 10 s, with a different set presented in each of the 36 blocks). The distractor task in the pilot was word-search puzzles, but resulting showup performance was higher than expected, so this new distractor task was meant to create some interference and to be somewhat more ecologically valid, as eyewitnesses would see other faces during the retention interval between crime and ID procedure.
3. They read a warning that the upcoming showup or lineup may or may not contain the target shown previously in that block^42^.
4. Participants made an identification decision for a TP or TA showup, biased lineup, or fair lineup, all of which contained faces with neutral expressions.
5. They entered their confidence in this decision on a 0–10 scale, with 0 = 0% confident in the decision, and 10 = 100% confident in the decision.

We randomly assigned participants to see all showups, all biased lineups, or all fair lineups. Within each of these conditions, the order of faces was randomized, and encoding time and target presence were counterbalanced across the 36 blocks.

### Results

**Fig. 4:**
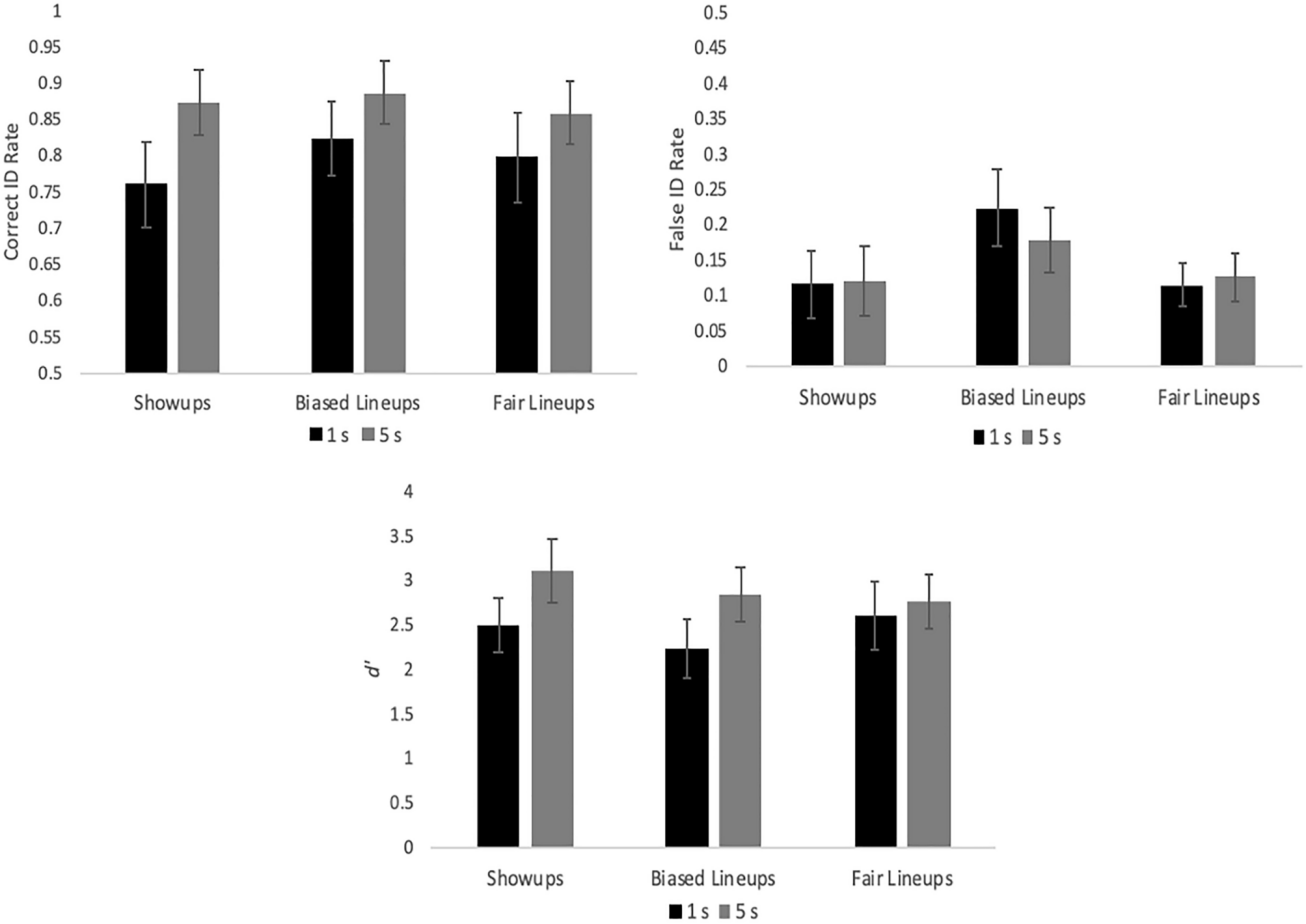
Correct ID rate (top left), false ID rate (top right), and discriminability (bottom), with 95% confidence interval bars, from Experiment 1.

We applied a mixed ANOVA model with our two independent variables (encoding time [within] and ID procedure [between], and their interaction) to each of our three ID measures separately (correct ID rate, false ID rate, and *d*′), controlling for Type I error rate with Bonferroni correction of alpha (0.05/3 = 0.0167). Starting with *d*′ (Fig. 4, bottom), there was no interaction and no effect of ID procedure, but longer encoding time increased discriminability, *F*(1, 133) = 16.32, *p* < 0.001, *η*_*p*_^2^ = 0.109. This difference in *d*′ is supported by ROC analysis (Fig. 1, upper-right panel): longer encoding time yielded greater discriminability (pAUC = 0.102) compared to our shorter encoding time (pAUC = 0.089), *D* = 6.72, *p* < 0.001. A similar pattern arose for correct ID rate, such that there was no effect of ID procedure and no interaction, but longer encoding time increased correct ID rate (Fig. 4, top left), *F*(1, 133) = 29.06, *p* < 0.001, *η*_*p*_^2^ = 0.178. Lastly, for false ID rate (Fig. 4, top right) there was no interaction and no effect of encoding time, but there was an effect of ID procedure, such that false ID rate was higher for biased lineups compared to showups and fair lineups, *F*(2, 133) = 6.20, *p* = 0.003, *η*_*p*_^2^ = 0.085.

**Fig. 5:**
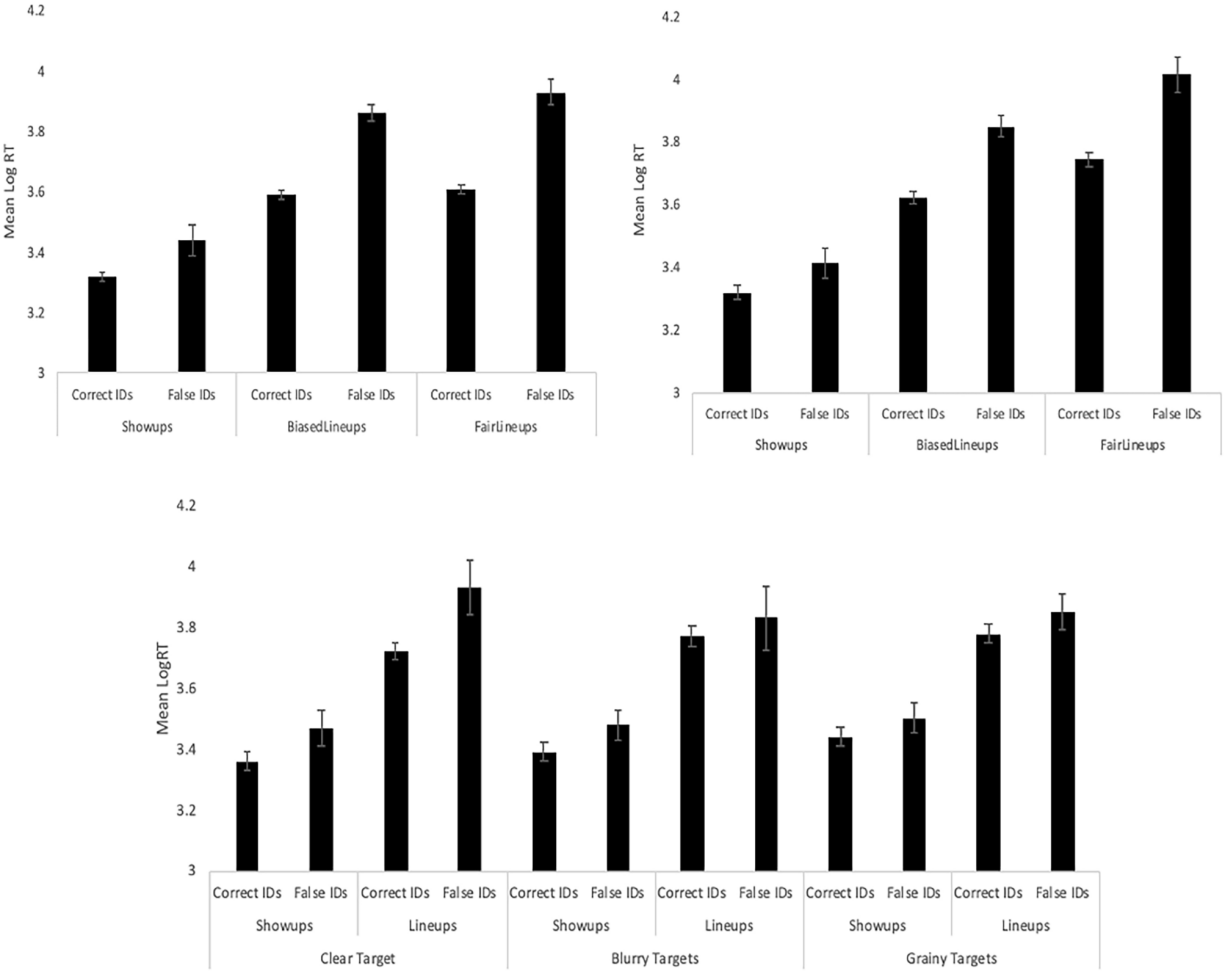
Mean log suspect ID time (with 95% confidence interval bars) from Experiment 1 (top-left), Experiment 2 (top-right), and Experiment 3 (bottom).

Turning to RT (range from Mdn 1902 ms [correct IDs for showups]—Mdn 8310 ms [false IDs from fair lineups]), after log transform and outlier deletion we applied an ANOVA model to assess effects of encoding time, ID procedure, and response (correct vs false ID). There was no effect of encoding time, *F*(1, 2687) = 0.82, *p* = 0.36, so we collapsed over it in Fig. 5 (top left panel). Correct IDs were faster than false IDs, *F*(1, 2687) = 347.96, *p* < 0.001, *η*_*p*_^2^ = 0.115, and there was an effect of ID procedure, *F*(2, 2687) = 343.43, *p* < 0.001, *η*_*p*_^2^ = 0.204, such that IDs from showups were faster than IDs from biased and fair lineups (which did not differ) based on Tukey HSD. There was no 3-way interaction, no interaction between encoding time and ID procedure, or between encoding time and response. There was an interaction between ID procedure and response, *F*(2, 2687) = 21.40, *p* < 0.001, *η*_*p*_^2^ = 0.016, such that correct IDs were faster than false IDs across all three ID procedures, but this difference was much greater for lineups compared to showups (Fig. 5, top left).

These results partially supported our hypothesis that ID speed would be slower for fair lineups compared to both showups and biased lineups. IDs were indeed slower for fair lineups compared to showups, but ID speed was equivalent between fair and biased lineups. This equivalence could be due to the suspect not standing out from our biased lineups, as all fillers still matched broad characteristics of the target, therefore participants would still need to scan over the faces before making an ID.

**Fig. 6:**
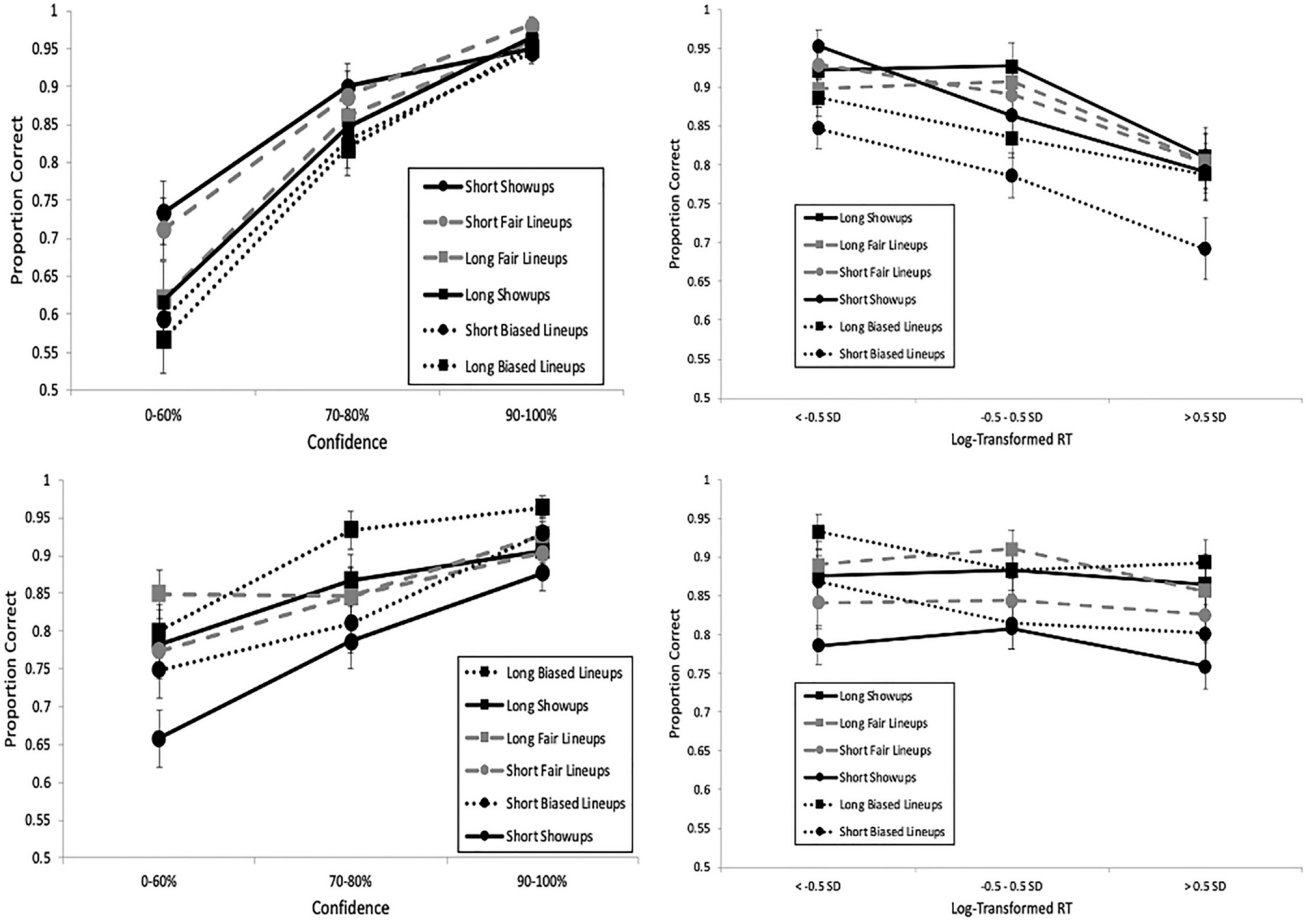
CAC (left panels) and RAC curves (right panels), with standard error bars, from Experiment 1. In the top row, proportion correct is based on identifications (correct/[correct + false IDs]), and in the bottom row it is based on rejections (correct rejections/[correct rejections + incorrect rejections]). “Long” indicates an encoding time of 5 s; “Short” indicates an encoding time of 1 s.

Moving on to the CA and RTA relationship, Fig. 6 (left) depicts the CAC and RAC curves (see supporting statistics in Table 2). As expected, there was a strong CA relationship for IDs (top left) regardless of encoding time, and the CA relationship for rejections (bottom left) was much flatter. As for ID procedure, there was a strong CA relationship (for IDs) for showups as well as lineups, in contrast to our prediction. The RTA relationship for IDs (upper right of Fig. 6) also remained strong regardless of memory strength. However, the RAC curves for IDs and rejections are flatter than the CAC curves, which provides additional support for confidence as a more important reflector of accuracy than RT. The flattest curves in the lower right of Fig. 6 represent a lack of RTA relationship for rejections. Moreover, mixed model analysis followed a similar pattern as the pilot, such that the confidence model performed the best, *F*(1, 5495) = 335.32, *p* < 0.001, relative to both the raw RT model, *F*(1, 5495) = 2.30, *p* = 0.129, and the LogRT model, *F*(1, 5495) = 20.94, *p* < 0.001.

**Fig. 7:**
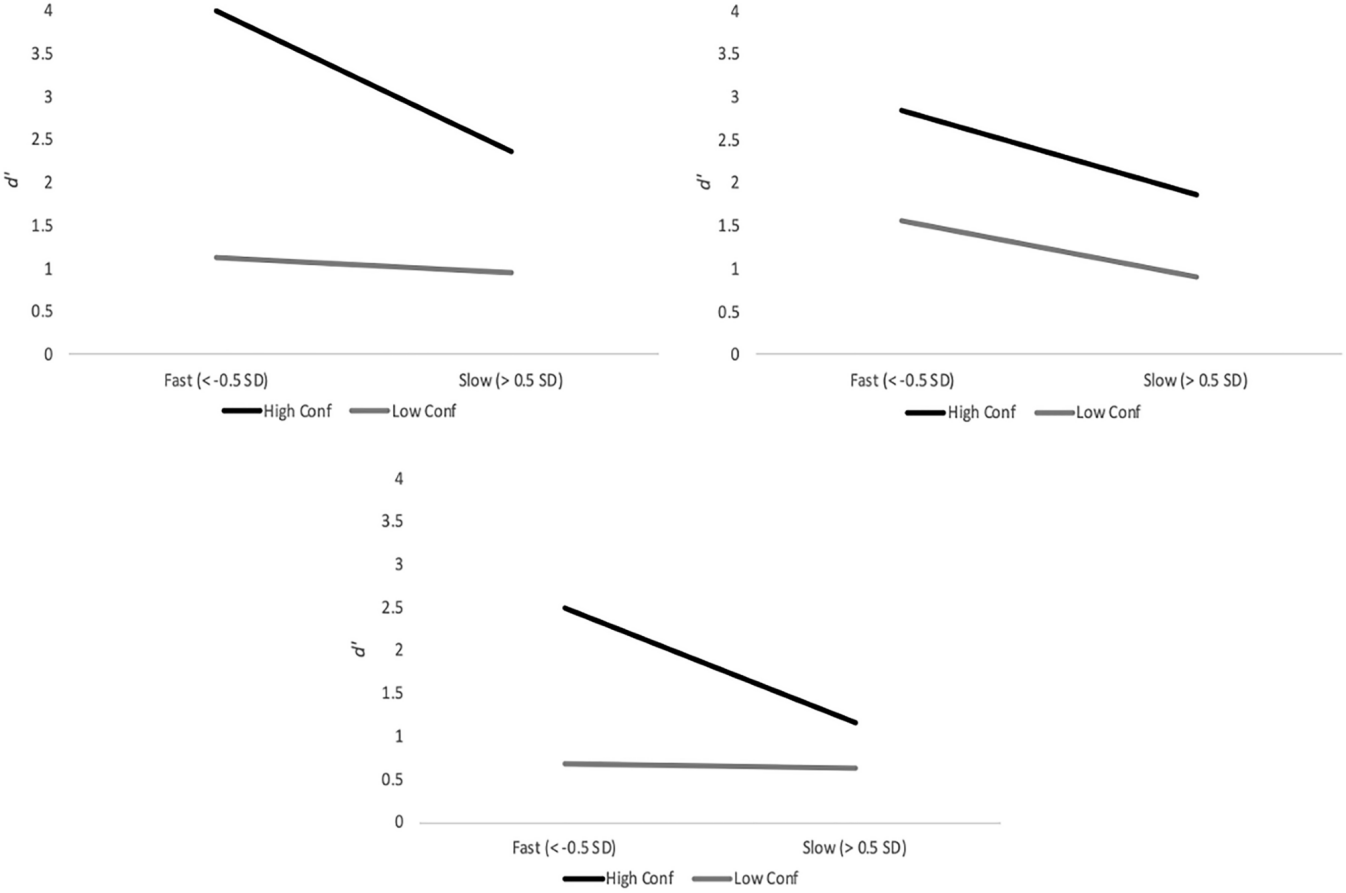
The value of confidence versus response time as reflectors of discriminability, from E1 (top left), E2 (top right), and E3 (bottom). Bars are 95% confidence intervals.

Another way of assessing the relative value of confidence versus RT as reflectors of accuracy is to compare the extremes. Figure 7 (top left panel) portrays high versus low confidence against fast versus slow IDs. To compare these *d*′s inferentially, we used the *G* statistic^43,59^, which can be treated like a *z*-score (all *p*-values are based on a two-tailed test). As shown in the figure (top left panel), fast IDs were more accurate than slow IDs when all were supported by high confidence (*G* = 5.48, *p* < 0.001), but RT had no effect on *d*′ at low confidence (*G* = 1.05, *p* = 0.15). In contrast, high confidence IDs were more accurate than low confidence IDs regardless of speed (slow, *G* = 6.91, *p* < 0.001; fast, *G* = 11.88, *p* < 0.001). It is also apparent that the combination of fast and highly confident produces the highest *d*′, which illustrates the increased value of having both reflector variables available to evaluate an ID.

### Discussion

There are three primary findings from this experiment. First, we found that confidence in IDs, and to a lesser extent confidence in rejections, could be used to indicate accuracy, but RT had no value for rejections and was a weaker reflector of ID accuracy compared to confidence. Second, the best case scenario for indicating accuracy was when high confidence was paired with fast RT. Third, ID speed was indicative of guilt for both showups and lineups, but this effect was much larger for lineups (Fig. 5). To our knowledge, this effect has not been shown before in the eyewitness literature, and we will attempt to replicate it with our next two experiments. There was no difference in discriminability between showups and lineups, but this was not surprising based on other within-subjects eyewitness ID designs^56^. The task could also still be too easy, thereby boosting showup performance to the level of the lineup, which we attempted to address in Experiments 2 and 3.

## Experiment 2

We utilized the same stimuli and procedure as E1 except we modified the distractor task again to make it more difficult. Instead of participants making attractiveness ratings for six computer-generated faces for one minute, we extended it to two minutes for 60 real faces. We expected this change to increase retroactive interference, thereby increasing task difficulty and more closely mimicking the real-world situation of an eyewitness encountering multiple people between a crime and ID procedure. See pre-registration here: https://osf.io/xpn37/?view_only=3ad28460f8fc450c8bb8a6aac7f0c803

### Method

#### Participants

Being that we repeated our design from E1 in this experiment, we had the same sample size goal from the same power analysis (at least 90 participants, or at least 30 per between-subjects condition). We ended up with *N* = 93 participants across three universities: the same two as provided data in E1, as well as another small university located in the northeast U.S. Each university collected data for the full experimental design. The demographic breakdown of the sample is as follows: (a) sex: 74% female; (b) age: 83% between 18 and 24, 9% between 25 and 30, and 8% between 30 and 40; (c) race/ethnicity: 64% Caucasian, 11% Hispanic or Latino, 11% African-American, 6% Asian or Indian, and 8% other or chose not to report.

#### Stimuli, design, and procedure

This experiment featured the same design and procedure as E1, except there were a few changes to the distractor task, as follows: (a) increased retention interval from 1 to 2 min, (b) attractiveness ratings for real faces from the Face Research Lab London Set^60^ rather than computer-generated FACES, and (c) increased number of faces from 6 to 60 (one every 2 s for the 2 min). The 60 distractor faces for each block were randomly selected (with replacement) from a large pool. They were never presented at encoding as targets or at retrieval as showups or in lineups.

### Results

**Fig. 8:**
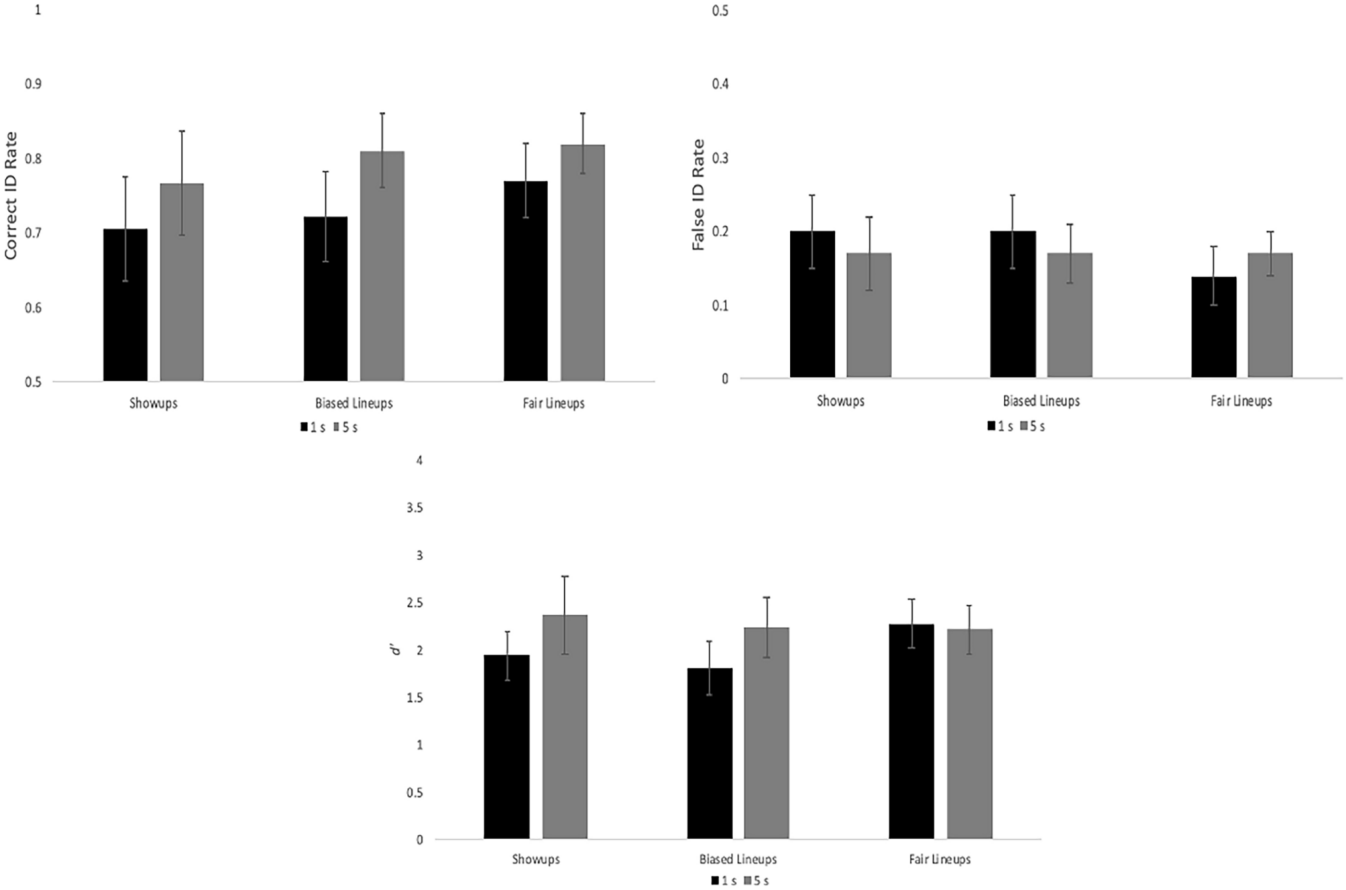
Correct ID rate (top left), false ID rate (top right), and discriminability (bottom), with 95% confidence interval bars, from Experiment 2.

To confirm an increased level of difficulty, *d*′ was lower (2.14) compared to E1 (2.67), *G* = 5.79, *p* < 0.001. A new mixed ANOVA model containing encoding time (within), ID procedure (between), and their interaction was applied separately to correct ID rate, false ID rate, and *d*′, again with Bonferroni correction to alpha (0.05/3 = 0.0167). There was no interaction and no effect of ID procedure for any measure. Instead all of the action was with encoding time. Longer encoding boosted correct ID rate (Fig. 8, top left), *F*(1, 90) = 13.69, *p* < 0.001, *η*_*p*_^2^ = 0.132, and *d*′ (Fig. 8, bottom), *F*(1, 90) = 7.80, *p* = 0.006, *η*_*p*_^2^ = 0.080, but had no effect on false ID rate (Fig. 8, top right), *F*(1, 90) = 0.33, *p* = 0.57, *η*_*p*_^2^ = 0.004. See also Fig. 1 (lower left panel) that shows the effect of encoding time on empirical discriminability: long (pAUC = 0.111) > short encoding (pAUC = 0.090), *D* = 3.41, *p* < 0.001.

Turning to RT (range from Mdn 1874 ms [correct IDs for showups]—Mdn 11,288 ms [false IDs from fair lineups]), after log transform we conducted an ANOVA to investigate the effects of encoding time, ID procedure, and response (correct vs false ID). Figure 5 (top right) depicts the results collapsed over encoding time because, as in E1, there was no effect of encoding time on log RT, and it did not interact with ID procedure or response. There was an effect of ID procedure, *F*(2, 1669) = 373.94, *p* < 0.001, *η*_*p*_^2^ = 0.309, and response, *F*(1, 1669) = 169.46, *p* < 0.001, *η*_*p*_^2^ = 0.092, as well as an interaction, *F*(2, 1669) = 11.44, *p* < 0.001, *η*_*p*_^2^ = 0.014. As shown in the figure, correct IDs were faster than false IDs, and Tukey HSD revealed that showup IDs were faster than biased lineup IDs, which in turn were faster than fair lineup IDs. As for the interaction, it was due to correct IDs being much faster than false IDs from lineups compared to showups.

**Fig. 9:**
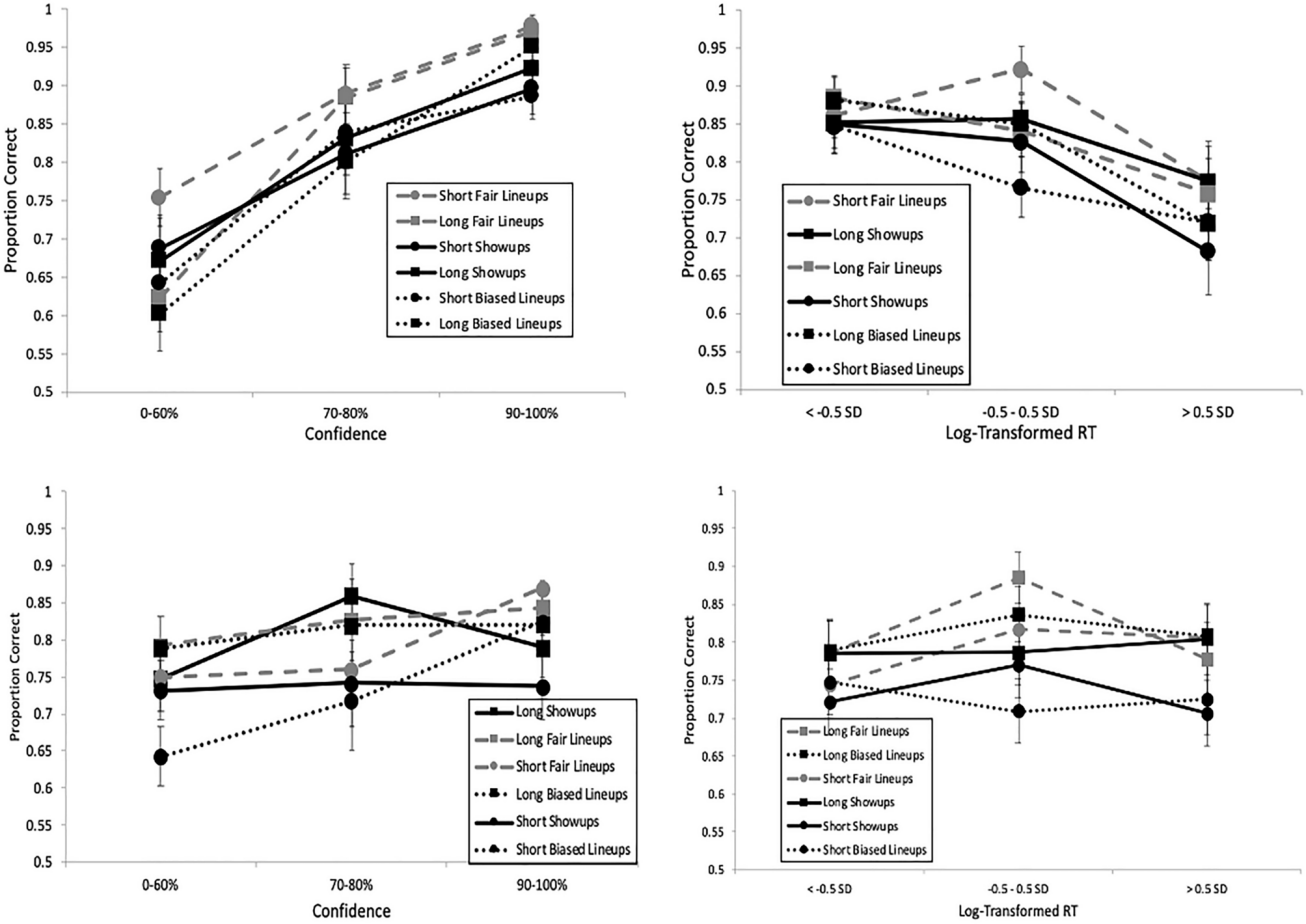
CAC (left panels) and RAC curves (right panels), with standard error bars, from Experiment 2. In the top row, proportion correct is based on identifications (correct/[correct + false IDs]), and in the bottom row it is based on rejections (correct rejections/[correct rejections + incorrect rejections]). “Long” indicates an encoding time of 5 s; “Short” indicates an encoding time of 1 s.

Next we investigated the CACs and RACs (Fig. 9). As in E1, there was a strong CA relationship (top left) and RTA relationship (top right) for IDs across encoding time and ID procedure (top left), with the RAC curves flatter than the CAC curves (see statistics in Table 2). All curves for rejections (bottom) are again flatter than curves for IDs (top). Mixed model analysis also revealed the strongest relationship for confidence, *F*(1, 3578) = 353.67, *p* < 0.001, over both raw RT, *F*(1, 3578) = 241.34, *p* < 0.001, and LogRT, *F*(1, 3578) = 201.42, *p* < 0.001.

Lastly, the extremes of confidence and ID speed are shown in Fig. 7 (top right), and the pattern is somewhat different from E1. As before, fast IDs were more accurate than slow IDs at high confidence (*G* = 4.17, *p* < 0.001), but now this pattern is also present at low confidence (*G* = 3.71, *p* < 0.001). In replication of E1, RT had no effect on the superiority of high over low confidence (slow, *G* = 3.70, *p* < 0.001; fast, *G* = 5.38, *p* < 0.001). The best performance remained with fast IDs supported by high confidence.

### Discussion

We accomplished our goal of increased task difficulty compared to E1, but still found no difference in *d*′ among our three ID procedures. We speculate about some theoretical rationale for this null effect in the General Discussion. More importantly, we replicated our three primary findings from E1: (a) RT was indicative of accuracy for both showups and lineups, but this effect was much larger for lineups, (b) confidence was a more valuable reflector of accuracy than RT overall based on the CAC and RAC curves, and (c) the highest discriminability came from fast IDs supported by high confidence. In our third and final experiment we attempted to extend these effects to a different manipulation of memory strength.

## Experiment 3

**Fig. 10:**
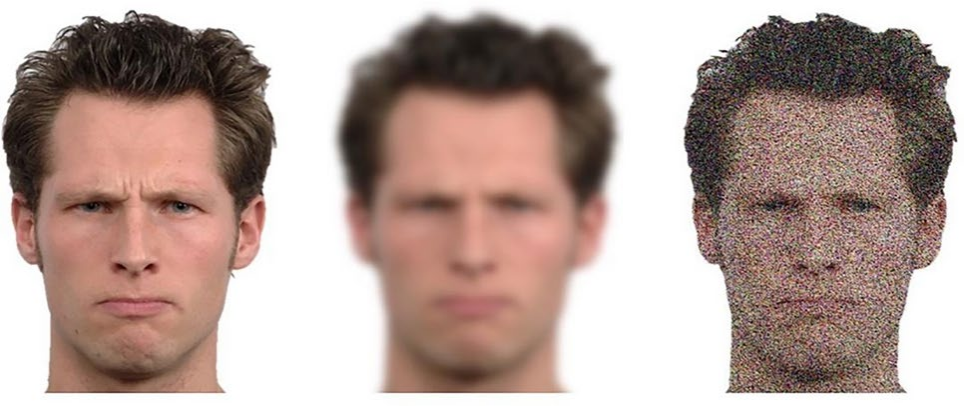
Example target images from Experiment 3: original (left), blurry (middle), and grainy (right).

In our final experiment we sought to extend our investigation to a different manipulation of memory strength: target image quality rather than target encoding time. We compared a clear image of the target with both a blurry and grainy version (Fig. 10). Pilot testing revealed that clear images yielded the best performance and grainy images created the worst performance, with blurry images in between. See our pre-registration here: https://osf.io/yuzes/?view_only=697e26a78aa74e06a975389bfa0b7cd8

### Method

#### Participants and design

The same universities as in E2 contributed participants (*N* = 175) for this final experiment, with the same sample size goal of 90. Our final sample size exceeded this goal because we continued to collect data until the end of semester, resulting in more participants than expected. The demographic breakdown of the sample is as follows: (a) sex: 79% female; (b) age: 89% between 18 and 24, 7% between 25 and 30, and 4% between 30 and 40; (c) race/ethnicity: 71% Caucasian, 9% African-American, 8% Hispanic or Latino, 7% Asian or Indian, and 5% other or chose not to report. We again had a 2 × 3 × 2 mixed design, but with a slight change to two of our independent variables. First, we dropped the biased lineups because they yielded similar results as our fair lineups, probably due to the fact that the suspect did not stand out as he would from an extremely biased lineup (i.e., our fillers all still matched the broad description of the perpetrator, just not in the details). This changed our previously 3-level ID procedure variable to two levels (showups, fair lineups). Additionally, we changed our memory strength variable from two encoding times (1 vs. 5 s) to three levels based on image quality (Fig. 10): clear, blurry, grainy. These changes resulted in a 3 (grainy versus blurry versus normal target at encoding, within subjects) × 2 (showup vs. lineup, between subjects) × 2 (TP vs. TA, within subjects) factorial design.

#### Stimuli and procedure

We utilized new targets and created new lineups, all with images from several face databases: Radboud Faces Database^38^, Face Place Tarr Lab^61^, KDEF^37^, Park Aging Mind Lab Face Database^62^, Meissner Face Database^63^, FACES^64^, Multi-PIE^65^, and ADFES^36^. We followed the same procedure as described in supplemental material for creating fair lineups. In terms of the experimental procedure, participants took part in 36 study-distractor-test blocks, with each target presented for 1 s, and the same distractor as E2.

### Results

**Fig. 11:**
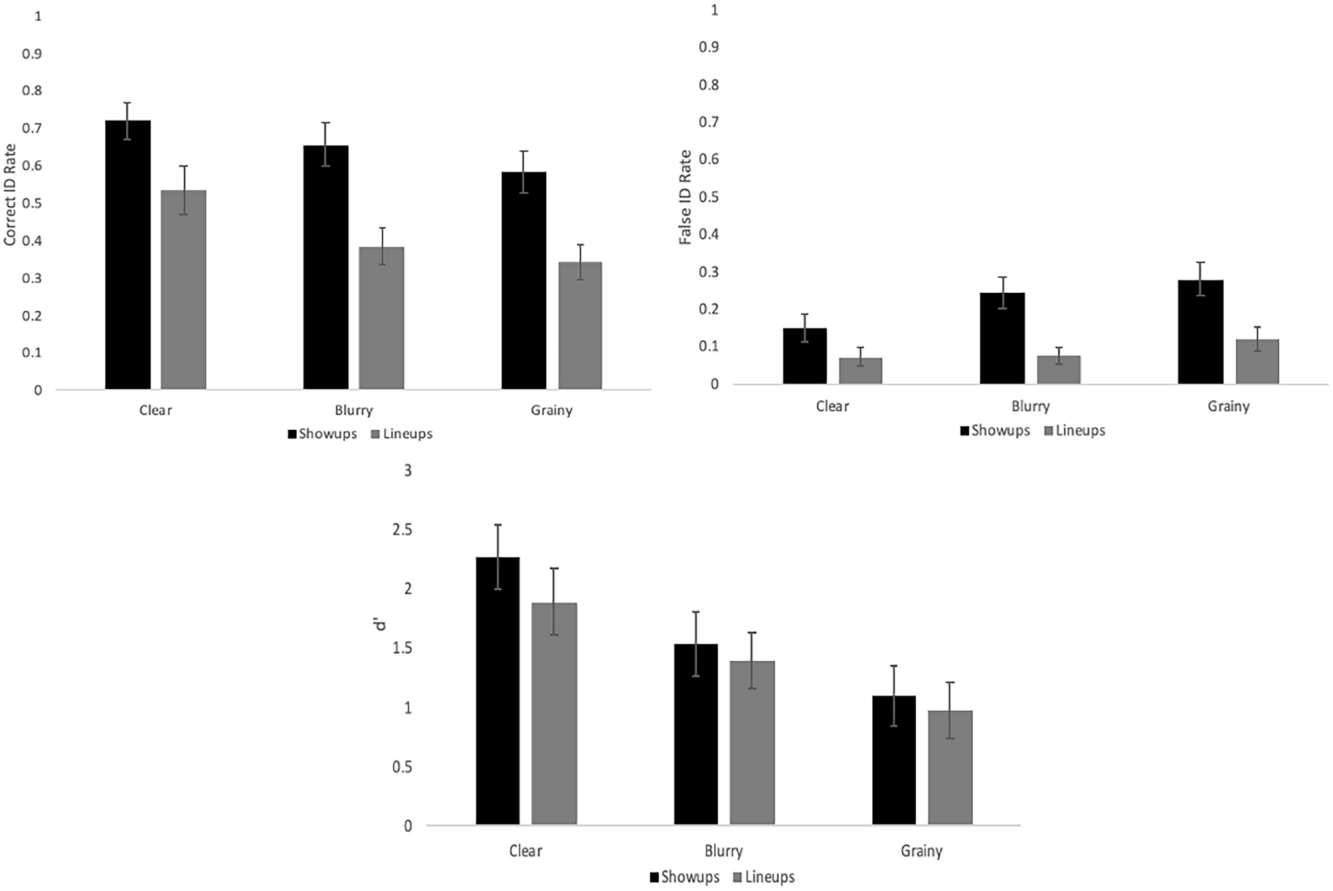
Correct ID rate (top left), false ID rate (top right), and discriminability (bottom), with 95% confidence interval bars, from Experiment 3.

See Fig. 11 for these results. A separate mixed ANOVA model with target quality (within), ID procedure (between), and their interaction was applied separately to correct ID rate, false ID rate, and *d*′, with Bonferroni correction to alpha (0.05/3 = 0.0167). Starting with ID procedure, both correct ID rate (*F*(1, 174) = 52.33, *p* < 0.001, *η*_*p*_^2^ = 0.231) and false ID rate (*F*(1, 174) = 50.91, *p* < 0.001, *η*_*p*_^2^ = 0.226) were higher for showups over lineups, but there was no difference in *d*′ (*F*(1, 174) = 2.60, *p* = 0.11, *η*_*p*_^2^ = 0.015). As for target quality, there was an effect of correct ID rate (*F*(2, 348) = 33.35, *p* < 0.001, *η*_*p*_^2^ = 0.161), false ID rate (*F*(2, 348) = 18.11, *p* < 0.001, *η*_*p*_^2^ = 0.094), and *d*′ (*F*(2, 348) = 44.42, *p* < 0.001, *η*_*p*_^2^ = 0.203). As shown in Fig. 1 (bottom right panel), clear images yielded the highest discriminability (pAUC = 0.107), followed by blurry (pAUC = 0.076) then grainy images (pAUC = 0.056). All pairwise differences were significant: (a) clear versus blurry, *D* = 9.20, *p* < 0.001; (b) clear versus grainy, *D* = 14.31, *p* < 0.001; and (c) blurry versus grainy, *D* = 6.62, *p* < 0.001. The interaction between ID procedure and target quality was non-significant for both correct ID rate (*F*(2, 348) = 2.33, *p* = 0.10) and *d*′ (*F*(2, 348) = 0.82, *p* = 0.44), and was only significant for false ID rate (*F*(2, 348) = 6.11, *p* = 0.002, *η*_*p*_^2^ = 0.034). This interaction can be interpreted as showups having higher false ID rate than lineups across all three levels of quality (clear, blurry, grainy), but the difference in false ID rate between showups and lineups is much larger (0.16) for blurry and grainy images compared to clear images (0.07).

We next analyzed the log-transformed RT data (raw range from Mdn 2100 ms [correct IDs for showups]—Mdn 8233 ms [false IDs from lineups]) with an ANOVA model containing effects of target quality, ID procedure, and response (correct vs false ID), as well as their interactions. There was an effect of ID procedure (*F*(1, 2183) = 544.89, *p* < 0.001, *η*_*p*_^2^ = 0.200) and response (*F*(1, 2183) = 39.05, *p* < 0.001, *η*_*p*_^2^ = 0.018), but no effect of target quality (*F*(2, 2183) = 1.16, *p* = 0.32). As shown in Fig. 5 (bottom panel), ID decisions were faster for showups compared to lineups, and correct IDs were faster than false IDs. All interactions were non-significant except for target quality and response, (*F*(2, 2183) = 3.37, *p* = 0.035, *η*_*p*_^2^ = 0.003), such that correct IDs were especially faster than false IDs when targets were clear.

**Fig. 12:**
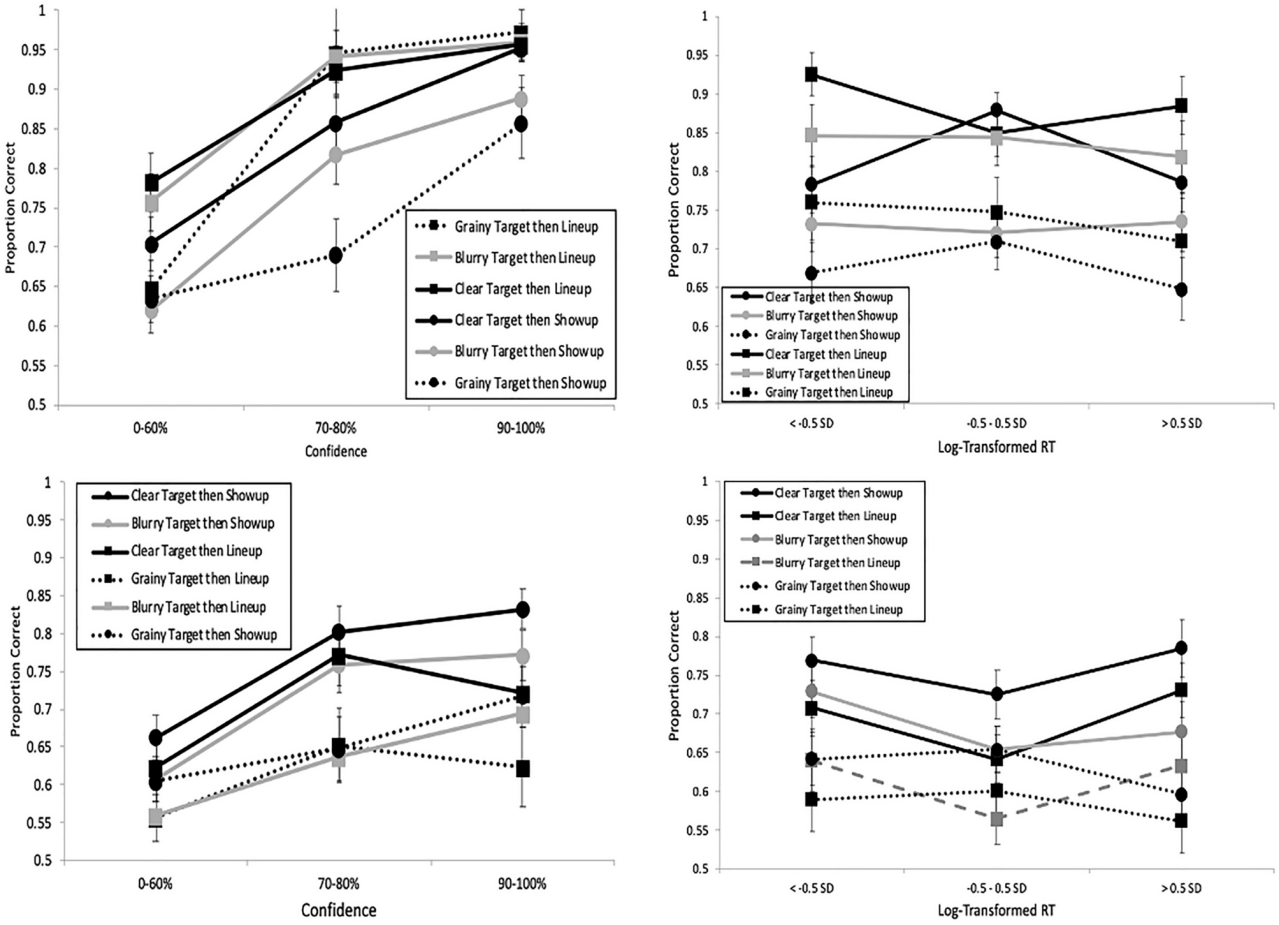
CAC (left panels) and RAC curves (right panels), with standard error bars, from Experiment 3. In the top row, proportion correct is based on identifications (correct/[correct + false IDs]), and in the bottom row it is based on rejections (correct rejections/[correct rejections + incorrect rejections]).

Next we analyzed the CAC and RAC curves (Fig. 12), with all of the following interpretations supported by statistics in Table 2. The curves providing the most information about accuracy were based on confidence in IDs (upper left panel), followed by confidence in rejections (lower left panel), and the RAC curves on the right provided little to no information about accuracy. This differs somewhat from our prior experiments that found a negative RTA relationship for IDs. However, the mixed model analysis supported the stronger relationship between confidence and accuracy, *F*(1, 6334) = 529.40, *p* < 0.001, relative to raw RT and accuracy, *F*(1, 6334) = 95.97, *p* < 0.001, or Log RT and accuracy, *F*(1, 6334) = 249.57, *p* < 0.001.

As for the extremes of confidence and RT (Fig. 7, bottom panel), the pattern from E1 returned, with RT having no value at low confidence (*G* = 0.39, *p* = 0.35), but at high confidence faster IDs were more accurate than slower IDs (*G* = 5.05, *p* < 0.001). In replication of E1, the advantage of high over low confidence was much larger for faster IDs (*G* = 10.77, *p* < 0.001) compared to slow IDs (*G* = 1.69, *p* = 0.046). In sum, this analysis aligns with our conclusion based on the CAC and RAC curves: confidence is a better reflector of accuracy than RT when alone. Moreover, we again supported the conclusion that having both types of information available (i.e., high confidence and fast RT) is ideal as it provides the most value.

**Fig. 13:**
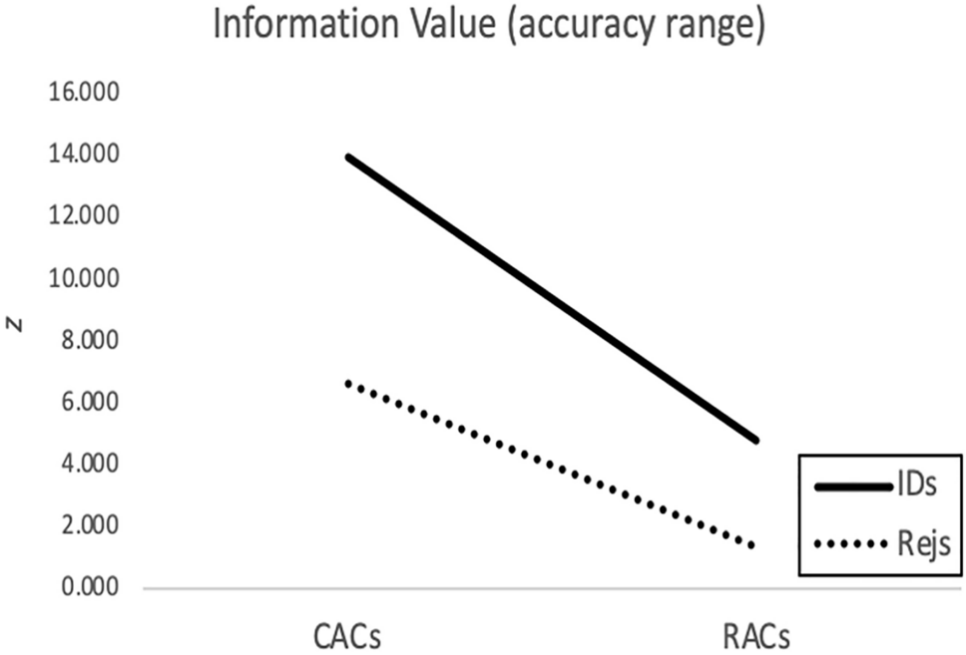
Informational value about accuracy derived from confidence versus response time, and identifications versus rejections. These are unweighted averages of the *z*-values in Table 2 across all experiments. IDs = identifications, Rejs = rejections.

In order to reinforce this conclusion, Fig. 13 combines the statistics in Table 2 across all experiments to depict the overall informational value of CACs versus RACs relative to IDs versus rejections. For decades the literature has consistently shown that confidence in IDs is far more informative than confidence in rejections^1^. Here we compare this advantage of IDs over rejections to the advantage of CACs over RACs, in terms of accuracy range. The conclusion is clear: if evaluated independently, confidence is a more informative reflector of accuracy compared to RT for both IDs and rejections, but especially for IDs.

### Discussion

With a different manipulation of memory strength (target image quality rather than encoding time), we replicated our three key findings from the first two experiments. First, given a clear target, RT was again indicative of accuracy, and this effect was still larger for lineups over showups. Second, confidence remained a better reflector of accuracy than RT. Third, the best reflector of accuracy was when fast IDs were combined with high confidence. We expand upon these findings below.

## General discussion

Across four experiments, we found that the CA relationship was stronger for IDs over rejections, and remained strong across levels of target memory strength and different ID procedures (showups, lineups). Confidence was a better reflector of accuracy than was RT, which is in agreement with basic old/new recognition memory research^9^. Moreover, we found mixed support for the pristine conditions hypothesis^1^, which predicts that fair lineups should yield a stronger CA relationship than biased lineups or showups. We found this pattern in E1 but typically showups and biased lineups produced a strong CA relationship. In terms of our estimator variable, memory strength had little to no impact on the CA relationship. We conclude that a more nuanced explanation for the CA relationship may be needed, which is in line with other recent studies finding exceptions to the pristine conditions hypothesis^16^.

In terms of RT, we supported an information accumulation model^32^, as correct IDs were faster than false IDs. However, we did not fully support memory strength-based differences in RT predicted by both the diffusion model^7^ and SDT^34^, such that correct IDs were not faster with stronger target memory. This may be due to our very short retention intervals (i.e., 1–2 min), which could have limited the strength of our manipulations, thus even after seeing a face for just 1 s, RT was still fast when accurate. In addition, Ratcliff^7^ found that greater memory strength corresponded with faster RT only when memory strength was manipulated within Ss. With our hybrid design (ID procedure always tested between subjects), we cannot distinguish between RT effects based on changes in memory strength versus potential differences in response threshold between participants or across blocks.

Our results established that the combination of high confidence and fast RT is most indicative of accuracy^3^. When an ID is collected in line with best-practice procedures^4^, this information is readily available. However, because these practices are not always followed, many cases go to trial where immediate confidence or RT is unknown. Triers of fact would benefit to know how confidence, RT, as well as the combination of these two variables relate to eyewitness accuracy.

With several methods, especially CAC and RAC curves, we found confidence to be a better reflector of accuracy than RT, which could be a result of differences in metacognitive processing. We acknowledge that confidence judgments can be generated via unconscious metacognitive beliefs^66–68^, but these are beyond the scope of this paper. For an eyewitness, a confidence judgment is primarily a conscious indication of the strength of memory match (target ID) or memory mismatch (rejection), but ID decision speed does not align with just one cognitive process. An eyewitness’s decision speed includes several additional cognitive and non-cognitive processes, such as scanning faces in a lineup, comparing each face with the memory of the target, deciding whether to make an ID or rejection decision, and then finally making that decision (e.g., with a spoken response or button press). It is understandable that this more diffuse process would not calibrate as precisely with accuracy, which like confidence is based on memory match. We recommend more research on the cognitive processes underlying both the CA and RTA relationship to better understand the CA advantage.

## Limitations

Our multi-block design is not an adequate representation of a single event witnessed by an eyewitness. Regardless, it is a commonly used paradigm to provide sufficient statistical power^54,56^ and we replicated several patterns from the literature in terms of discriminability, the CA relationship, and the RTA relationship. One pattern that we did not replicate is the typical discriminability advantage of fair lineups over showups^23^. As noted above, we anticipated this possibility because other studies with a similar paradigm have also not found this difference^56^. We can only speculate as to the potential mechanisms explaining this null finding, but one possibility is based on the Diagnostic Feature Detection theory (DFT) of eyewitness identification^69^. Briefly, this theory states that discriminability is increased by procedures that allow eyewitnesses to compare features across faces, thereby determining which features are diagnostic cues to the identity of the perpetrator. For example, if the perpetrator was a young Caucasian man with a dark beard, an eyewitness could identify an innocent man with a dark beard when presented as a showup (thereby reducing discriminability), but when this man is surrounded by similar bearded fillers in a fair lineup, the eyewitness is likely to discount the beard and instead focus on other aspects of the face (e.g., “no one here has the same blue eyes as the perpetrator, so I will reject this lineup”). Our multi-block paradigm may have allowed for this kind of process for showups, as our participants could determine across blocks that all faces shared certain characteristics (e.g., young Caucasian men). If a DFT-type process was utilized for showups, it would have theoretically boosted discriminability to the level of the lineup.

## Conclusions

Though we found some value in confidence and RT as reflectors of accuracy for showups, there are still reasons to prioritize lineups. Correct IDs were faster than false IDs across all ID procedures, but the difference was much more pronounced for lineups relative to showups. Our findings align well with current recommendations^4^: (a) prioritize lineups over showups, (b) collect immediate confidence after the eyewitness’s decision, and (c) video-record ID procedures in part to assess decision time. Evaluating eyewitness accuracy is best informed by the combination of confidence and decision time.

## Data Availability

The data are available on the Open Science Framework at the following links: Pilot: https://osf.io/wem46/files/osfstorage/6734fccdbf6f7e59fd8ea3ab. Experiment 1: https://osf.io/wem46/files/osfstorage/67350b3a07d3f52bed0de0a4. Experiment 2: https://osf.io/wem46/files/osfstorage/67351dfe09bb089f0f8ea446. Experiment 3: https://osf.io/wem46/files/osfstorage/67363156d31486b4c8d05dba
